# Natural IgM antibodies that bind neoepitopes exposed as a result of spinal cord injury , drive secondary injury by activating complement

**DOI:** 10.1186/s12974-017-0894-6

**Published:** 2017-06-19

**Authors:** Aarti Narang, Fei Qiao, Carl Atkinson, Hong Zhu, Xiaofeng Yang, Liudmila Kulik, V. Michael Holers, Stephen Tomlinson

**Affiliations:** 1 Department of Microbiology and Immunology, Medical University of South Carolina, 173 Ashley Ave, CRI 213, Charleston, SC 29425 USA; 2 Departments of Medicine and Immunology, University of Colorado School of Medicine, Denver, CO USA; 3 Ralph H Johnson VA Medical Center, Charleston, SC USA

**Keywords:** Complement, Spinal cord injury, Natural antibodies, IgM, Neoepitope, Therapy

## Abstract

**Background:**

Natural IgM antibodies (Abs) function as innate immune sensors of injury via recognition of neoepitopes expressed on damaged cells, although how this recognition systems function following spinal cord injury (SCI) exposes various neoepitopes and their precise nature remains largely unknown. Here, we investigated the role of two natural IgM monoclonal Abs (mAbs), B4 and C2, that recognize post-ischemic neoepitopes following ischemia and reperfusion in other tissues.

**Methods:**

Identification of post-SCI expressed neoepitopes was examined using previously characterized monoclonal Abs (B4 and C2 mAbs). The role of post-SCI neoepitopes and their recognition by natural IgM Abs in propagating secondary injury was examined in Ab-deficient Rag1−/− or wild type C57BL/6 mice using Ab reconstitution experiments and neoepitope-targeted therapeutic studies, respectively.

**Results:**

Administration of B4 or C2 mAb following murine SCI increased lesion size and worsened functional outcome in otherwise protected Ab-deficient Rag1−/− mice. Injury correlated with colocalized deposition of IgM and C3d in injured spinal cords from both mAb reconstituted Rag1−/− mice and untreated wild-type mice. Depletion of peritoneal B1 B cells, a source of natural Abs, reduced circulating levels of IgM with B4 (annexin-IV) and C2 (subset of phospholipids) reactivity, reduced IgM and complement deposition in the spinal cord, and protected against SCI. We therefore investigated whether the B4 neoepitope represents a therapeutic target for complement inhibition. B4-Crry, a fusion protein consisting of a single-chain Ab derived from B4 mAb, linked to the complement inhibitor Crry, significantly protected against SCI. B4-Crry exhibited a dual function in that it inhibited both the binding of pathogenic IgM and blocked complement activation in the spinal cord.

**Conclusions:**

This study identifies important neoepitopes expressed within the spinal cord after injury. These neoepitopes are recognized by clonally specific natural IgM Abs that activate complement and drive pathology. We demonstrate that these neoepitopes represent novel targets for the therapeutic delivery of a complement inhibitor, and possibly other payload, to the injured spinal cord.

**Electronic supplementary material:**

The online version of this article (doi:10.1186/s12974-017-0894-6) contains supplementary material, which is available to authorized users.

## Background

Traumatic spinal cord injury (SCI) results in severe debilitation, often with complete paralysis and loss of sensory function below the injury site. Following a primary mechanical insult, a secondary wave of cell death ensues, that is accompanied by ischemia, vascular damage, glutamate excitotoxicity, ionic dysregulation, and inflammation. The area of secondary injury emanating from the site of trauma represents a therapeutic target.

There is strong evidence that complement plays an important role in secondary SCI, and both the classical [1] and alternative [1, 2] pathways of complement activation have been implicated in propagating injury (reviewed in [3]). The alternative pathway functions as an amplification loop for the classical and lectin pathway of activation, although the role of the lectin pathway has not been investigated in SCI. Also, complement fragment levels have been found to be elevated in sera of spinal cord injury patients [4]. In an experimental model, it has been shown that complement activation and the propagation of SCI can be caused by pathogenic autoantibodies that arise following SCI-mediated activation of B cells [5, 6]. However, the role of naturally occurring pre-existing self-reactive antibodies (nAbs) produced by innate B1 B cells, and how complement is activated acutely after SCI, has not been investigated. Since SCI shares some pathophysiological characteristics with ischemia reperfusion injury (IRI), and since IRI is driven by natural IgM Ab-mediated activation of complement in some organs [7–10], we investigated a role for natural IgM Abs in propagating SCI.

Tissue ischemia exposes neoepitopes known as damage-associated molecular patterns (DAMPs) on the cell surface, and these neoepitopes are recognized by circulating natural IgM Abs that subsequently activate complement [11]. Some of these post-ischemic neoepitopes have been identified in the context of IRI and include non-muscle myosin [8], annexin IV [9], and various phospholipids [10, 12]. However, the nature of the neoepitopes exposed as a result of trauma on the injured spinal cord has not been investigated. Natural IgM Abs differ from adaptive immune Abs in that they are germline encoded, arise without exogenous antigen stimulation, and are produced by the B1 subset of B cells. B1 cells differ from conventional B2 cells by their phenotype, self-renewing capacity, and production of natural Abs. Although most natural IgM Abs are polyreactive, the subsets that initiate IRI are unique in that they recognize a specific set of epitopes or patterns and, importantly, are specific for neoepitopes on stressed or injured cells. Here, we investigate a pathogenic role for natural IgM Abs in promoting spinal cord injury, but it is important to note that natural Abs also have important physiological roles, such as protection from pathogens and the removal of cellular and molecular waste [13, 14]. Indeed, a monoclonal IgM Ab corresponding to a natural Ab specificity is currently undergoing clinical evaluation as a potential therapeutic for multiple sclerosis [15, 16].

We previously isolated two natural IgM mAbs from unmanipulated mice, B4 mAb that recognizes annexin IV and C2 mAb that recognizes a subset of phospholipids, and demonstrated that these mAbs specifically bind to the intestine [9], brain [10], and heart [17] tissue following ischemia and reperfusion. In this study, we make use of these mAbs to investigate neoepitope expression post-SCI.

## Methods

### IgM mAbs and annexin IV purification

Hybridomas expressing B4 and C2 IgM mAbs were isolated after fusion of splenocytes from unmanipulated C57BL/6 mice with SP2/0-AG14 myeloma cells, with screening of hybridomas for specificity to apoptotic cells [9]. B4 mAb has been shown to recognize annexin IV [9] and C2 mAb to recognize a subset of phospholipids [10]. F632 and D5 IgM mAbs recognize 4-hydroxy-3-nitrophenylacetyl [17] and cytokeratin-19 [9], respectively, and were used as control mAbs. The D5 mAb was isolated from the same panel of hybridomas from which B4 and C2 mAbs were isolated, and does not bind to apoptotic cells. The IgM mAbs were purified from culture supernatant by affinity chromatography using an agarose bead column conjugated with goat anti-mouse IgM (Sigma-Aldrich, St Louis, MO) as described [17]. Recombinant annexin IV, the B4 ligand, was expressed in 293 F cells and purified from culture supernatant as described [9].

### Injury model and treatments

Mice used in this study were female C57BL/6 mice and Rag1−/− mice on a C57BL/6 background, weighing between 18 and 22 g and between 6 and 8 weeks old, and obtained from Harlan Labs (Indianapolis, IN). Before the procedure, mice were anesthetized with ketamine/xylazine anesthesia (80 mg/kg/10 mg/kg, i.p.) and subjected to laminectomy T9-T12. Contusion injury was performed at T10 using an Infinite Horizon impactor (Precision Systems and Instrumentation, LLC, Lexington, Kentucky) at 80 Kdynes [18]. Sham control mice underwent laminectomy without contusion injury. Mice were kept on a temperature-controlled heating pad until the animals regained their mobility, after which they were placed back in a recovery cage on Alpha dri bedding. Mice were monitored, and bladders were voided twice daily until the bladder function was restored. For IgM mAb reconstitution experiments, Rag1−/− mice were randomized into different mAb treatment groups and a control group. Rag1−/− mice were injected i.v. with 100 μg C2, B4 or control mAbs 30 min after surgery. For therapeutic studies, animals were randomized into vehicle and B4Crry treatment groups and a single dose of 0.2 mg of protein was administered i.v. 30 min after injury. All procedures were approved by the Institutional Animal Care and Use Committee (IACUC) at the Medical University of South Carolina.

### B1 B cell depletion

Peritoneal B1 cell depletion was achieved by a modification of the method described by Peterson et al. [19]. Briefly, starting at 4 weeks of age, mice were administered progressively increasing amounts of distilled water, injected i.p. once per week over a 6-week period (2 injections each of 0.2, 0.5, and 1 ml). One group of mice received PBS injections as control. One day after the final injection, mice were used for procedures. For analysis of B1 B cell depletion, peritoneal cells were obtained by injecting 5 ml of ice cold PBS into the peritoneal cavity, gently massaging the cavity, and collecting lavage fluid containing peritoneal cells using a 22-gauge needle. Single-cell suspensions of these cells were incubated with CD16/32 for 5 min at 4 °C to block Fc receptor binding [19] and were then analyzed by flow cytometry using flourochrome-conjugated Abs: anti-mouse CD19 APC and CD45R (B220) FITC (eBioscience, San Diego, CA). Gate was placed on live (7-Aminoactinomycin D viable stain) peritoneal cells, and CD19^+^ IgM^hi^ B220^lo^ populations were quantified.

### Natural IgM levels in mouse serum

Blood was collected immediately following harvest of peritoneal cells, and serum prepared. Levels of anti-annexin IV and anti-phosphatidylcholine IgM Abs in mouse serum were quantified by ELISA using purified recombinant annexin IV (see above) and PC-BSA (Sigma) as capture reagents as previously described [10]. An ELISA kit was used to measure bound IgM in mouse serum according to the manufacturer’s instructions (Bethyl Laboratories, Montgomery, TX).

### Behavioral tests

Open-field assessment of locomotor function was done using the BMS scoring system with a scale of 0–9. The BMS scale is based on operational definitions of hind limb movement. Briefly, individual mice were simultaneously observed by two blinded investigators for a 4-min testing period, during which hind limb movements, trunk/tail stability, and forelimb-hind limb coordination were assessed and then graded according to published methods [20]. All mice subjected to contusion injury exhibited a BMS score of 0 immediately after injury. The grip walk test (also referred to as the foot fault task), modified from the grid walk/ladder rung walking task [21], assesses deficits in descending motor control and is a test of motor coordination [22]. The apparatus consists of a grid floor with the grid raised above the surface. Mice were placed at one end of the grid and monitored from the side as they walked across the grid. The number of hind limb grips instead of placement errors as the animal traverses the grid was scored. Each time the paw gripped the bar was counted and compared between different treatment groups. All analyses were performed by investigators blinded to the treatment groups.

### Tissue histology

Spinal cord tissue obtained from mice at 72 h, 7 days, or 21 days after injury was fixed in 10% formalin and embedded in paraffin. Seven-micrometer transverse sections were cut using a microtome and stained with standard hematoxylin and eosin stains for stereological analysis. Photographic documentation was made using Axio Vision Software (Carl Zeiss) at the resolution of 1024 × 1024 pixels. The hemorrhaged area and lesional tissue area was outlined using the measure tool from axiovision (refer to Additional file 1: Figure S2), and the extent of hemorrhage and lesion expressed as percentage of the total area per section. This analysis was done using 100-μm step sections across the length of 1 mm starting at 100 μm from the epicenter on each side [22].

### Immunohistochemistry and immunoflourescence

Spinal cord tissue was embedded in OCT on dry ice and frozen sections were fixed in acetone for 6 min, air dried, and then equilibrated in PBS. For paraffin sections, tissue was fixed in 10% formalin and processed by standard techniques. Deposition of the complement activation product, C3d, was detected using a goat anti-mouse C3d Ab (1:30, R&D Systems, Minneapolis, MN) with rabbit anti-goat IgG AlexaFluor-555 conjugate as secondary Ab (1:100, Invitrogen, Carlsbad, CA). IgM binding was detected using a goat anti-mouse IgM FITC conjugate (1:100, EMD Millipore, Billerica, Massachusetts). The images were taken using Olympus Fv10i Confocal microscope, images were processed using Adobe Photoshop CS6 software (Adobe System Inc., San Jose, CA, USA), and manipulations were limited to brightness/contrast functions. ImageJ and Fiji plugin (NIH) were used for analysis of images for fluorescence intensity. The intensity was measured and averaged from 100-μm step sections across a length of 1 mm, centered at the epicenter and starting at 100 μm from the epicenter on each side.

### Statistical analysis

Statistical differences between parametric data were assessed using one-way analysis of variance (ANOVA) test with Bonferroni’s multi-group comparison, and non-parametric data were compared with the Kruskal-Wallis test with Dunn’s comparison (Prism 5.0, GraphPad). Differences between data were considered statistically significant when *p* < 0.05.

## Results

### Specific IgM mAbs restore SCI in otherwise protected Rag1−/− mice

**Fig. 1:**
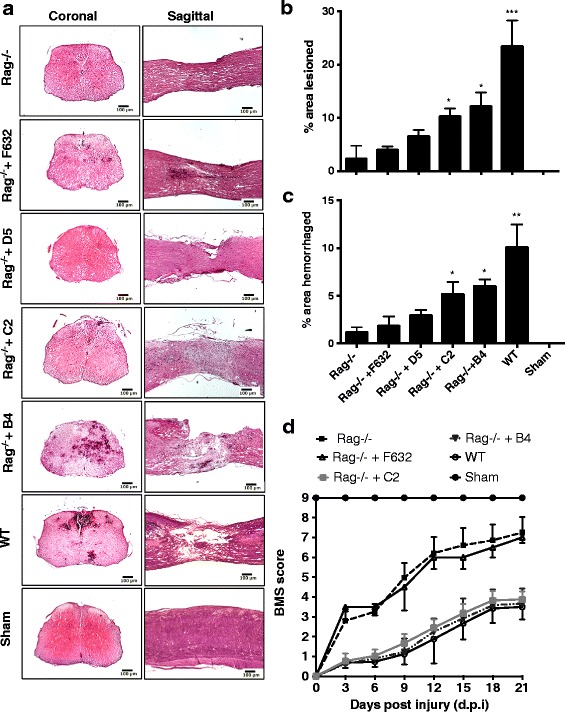
Specific IgM mAbs restore injury in otherwise protected Rag1−/− mice. **a** Representative H&E stained coronal and sagittal sections of WT mice, Rag1−/− mice, and Rag1−/− mice reconstituted with indicated mAb. As quantified in subsequent panels, histopathology revealed Rag1−/− mice were protected from injury, B4 and C2 mAbs restored injury in Rag1−/− mice, and control F632 and D5 mAbs had no significant effect on injury in Rag1−/− mice. **b** Lesion size determined from step sections obtained across 1-mm distance from the epicenter and expressed as percentage of total area of each section analyzed. Mean ± SE, ***p < 0.001, **p < 0.01, *p < 0.05 represent comparisons to Rag1−/−, *n* = 6 per group. **c** Hemorrhaged area determined from step sections obtained across 1-mm distance from the epicenter and expressed as percentage of total area of each section analyzed, Mean ± SE, ** *p* < 0.01, *p < 0.05 represent comparisons to Rag1−/−, *n* = 6 per group. **d** Open field 10-point BMS score to assess functional recovery over a 21-day period after SCI. A score of 9 represents no impairment of locomotor activity. Groups consisted of WTC57/bl6 and Rag1−/− mice, and Rag1−/− mice reconstituted with indicated IgM mAb. Mean ± SE, *p < 0.05, *n* = 6–8. Starting at day 3, significant differences were seen between Rag1−/− mice and Rag1−/− mice treated with control mAb vs. WT mice and Rag1−/− mice reconstituted with B4 and C2 mAbs

Recognition of ischemic tissue by natural self-reactive IgM promotes injury in various organs subsequent to reperfusion. To better understand the pathophysiology of inflammatory secondary damage following SCI, we investigated whether a similar IgM recognition system is involved in promoting secondary injury to the spinal cord after trauma. Antibody deficient Rag1−/− mice were protected from SCI, but injury outcomes worsened when Rag1−/− mice were injected with either B4 or C2 IgM mAb within 30 min of injury. We found significantly reduced lesion size and hemorrhaging at 3 days post-injury in Rag−/− mice after injury (Fig. 1a–c). Injection of specific natural Abs caused greater extent of injury in otherwise protected Rag−/− mice. Reconstitution of Rag1−/− mice with control F632 mAb or D5 mAb (Fig. 1a–c) did not alter outcome measures, demonstrating that the enhanced injury phenotype is neoepitope specific. We utilized one of these two specificities as controls for further studies. Compared to wild-type (wt) mice, Rag1−/− mice had significantly improved locomotor activity over a 21-day period post-injury (Fig. 1d). BMS measures in Rag1−/− mice were significantly worse upon reconstitution with B4 or C2 mAb, while injection of F632 mAb did not cause any significant changes.

### Analysis of IgM binding and C3 deposition following SCI

**Fig. 2:**
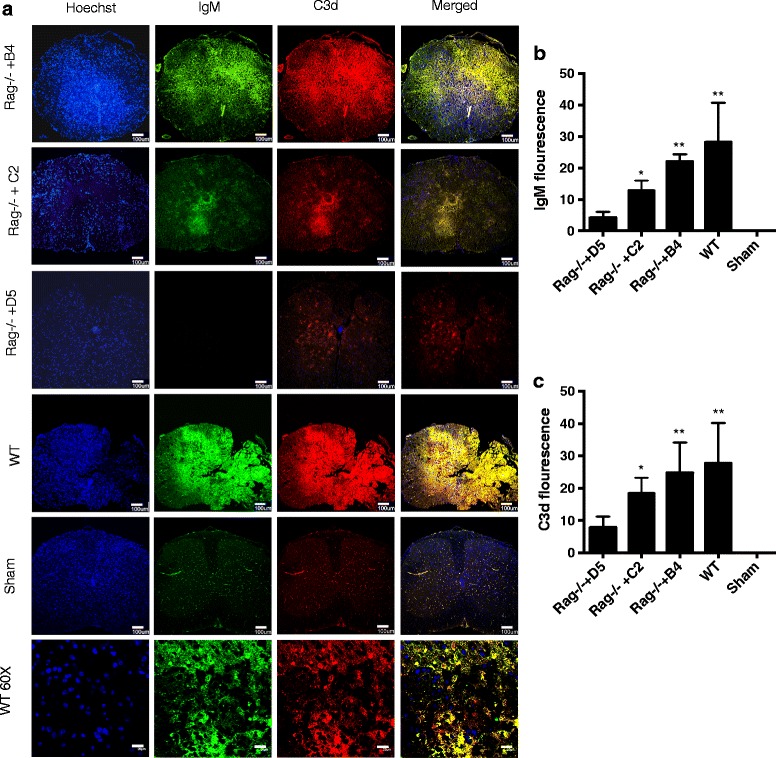
IgM binding and C3 deposition in injured spinal cords of wt and B4 mAb reconstituted Rag1−/− mice. Coronal step sections were obtained up to 0.5 mm each side from epicenter of contusion injury, 72 h after SCI. **a** Analysis of IgM and C3d deposition in spinal cords isolated from wt mice or Rag1−/− mice reconstituted with either B4 mAb or D5 mAb (control). Composite image (merged) indicates co-localization. Bottom panel shows high mag images of injured WT spinal cord. **b**, **c** Quantification of IgM binding and C3d deposition was done by measuring intensity from step sections across the length of 1 mm, centered at the epicenter and starting at 100 μm from the epicenter on each side. Mean ± SE, *p < 0.05, **p < 0.01, *n* = 6 per group. Scale bar = 100 μm

Complement has been shown to play a key role in modulating the progression of SCI, and we investigated the specificity of B4 mAb binding to the injured spinal cord together with analysis of complement activation. Spinal cord sections obtained from wt mice at the lesion site 3 days post injury demonstrated co-localization of IgM and the complement activation fragment C3d (Fig. 2). Co-localization of IgM and C3d was similarly demonstrated in Rag1−/− mice reconstituted with B4 mAb, but not in Rag1−/− mice reconstituted with control D5 mAb, correlating with the injury profiles shown above. Together with previous data showing an important role for complement in SCI, these results indicate specific IgM-mediated activation of complement is propagating injury.

### Peritoneal B1 B cell depletion reduces levels of circulating pathogenic natural IgM antibodies and provides protection in the acute phase of injury

**Fig. 3:**
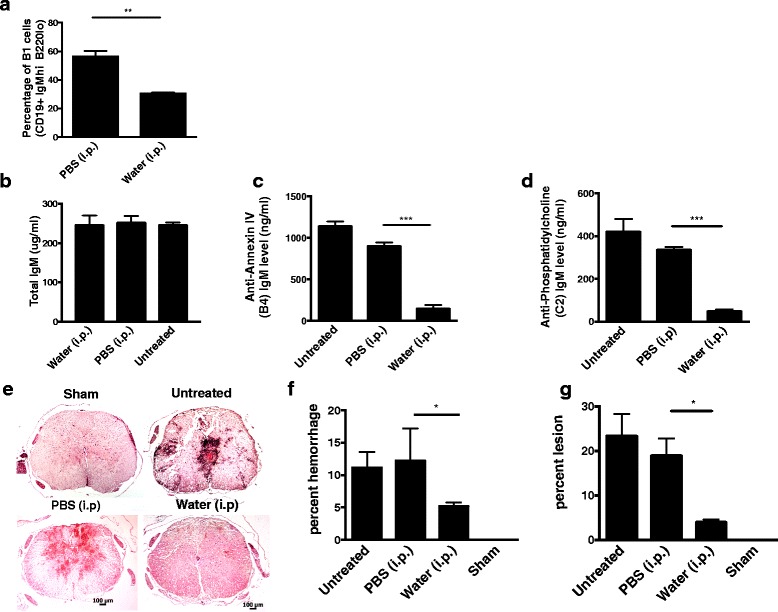
B1 B cell depletion reduces circulating levels of neoepitope-specific natural IgM Abs and protects against SCI. **a** Quantification of B1a B cell depletion by flow cytometry analysis of peritoneal cells following a 6-week hypotonic shock procedure, demonstrating depletion of peritoneal B1 B cells (B220^lo^CD5^+^). Mean ± SE, *p < 0.05, *n* = 3, and data is representative of three separate experiments. **b**–**d** Analysis of serum levels of total IgM, specific natural IgM antibodies with B4 mAb reactivity (annexin-IV) and C2 reactivity (phosphatidylcholine), determined by ELISA. Serum from mice treated with peritoneal injections of either PBS or water, as well as serum from WT mice, was analyzed. Mean ± SE, *p < 0.01, *p* < 0.001 respectively, *n* = 3–5. **e** Following B1 cell depletion or a control procedure, WT mice were subjected to SCI, and spinal cords isolated 72 h later for analysis. **f**, **g** Hemorrhaged area and lesion size and from step sections obtained across 1-mm distance, 0.5 mm from each side of the epicenter, and expressed as percentage of total area of each section analyzed. *p < 0.05, **p < 0.01, ***p < 0.001, *n* = 6 animals per group

**Fig. 4:**
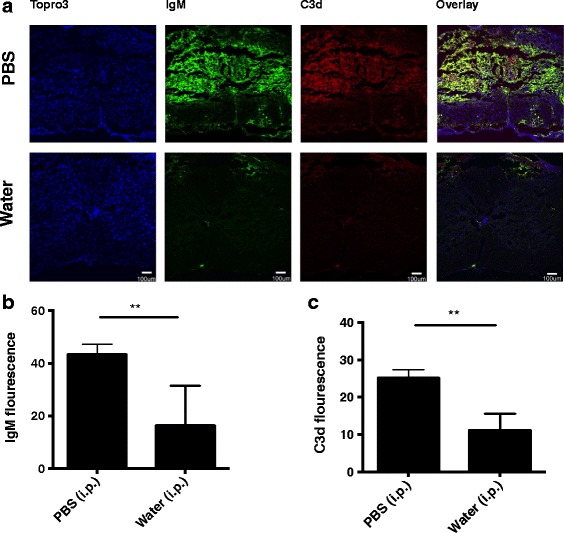
B1 B cell depletion reduced IgM deposition and complement activation in the injured spinal cord. **a** Immunofluorescence images of IgM and C3d deposition in cords isolated from control and B1 B cell depleted mice. Coronal step sections (100 um) were obtained across 1 mm from epicenter of contusion injury, and intensity measured and averaged. Composite image (merged) indicates co-localization. Representative images, *n* = 6. **b**, **c** Quantification of IgM binding and C3d deposition, respectively. Mean ± SE, **p < 0.01, *n* = 6

Natural IgM Abs are produced by innate like B1 B cells that are capable of self-renewal and are present in high numbers in the peritoneum. To confirm that the pathogenic effect in this model is mediated by natural Abs, we investigated the effect of peritoneal B1 B cell depletion on SCI in wt mice. B1 B cells were depleted using a hypotonic shock method involving intraperitoneal injection of water, and depletion was confirmed by flow cytometric analysis that demonstrated water treatment resulted in a significant reduction in CD19^+^ B220^lo^ peritoneal cells (Fig. 3a) (see also additional file Figure S2). As previously shown [19], peritoneal B cell depletion did not result in a reduction of total circulating IgM (Fig. 3b). However, peritoneal B1 B cell depletion did result in a significant decrease in circulating Abs of B4 and C2 specificities, further indicating the natural Ab status of anti-annexin IV and anti-phospholipid IgM mAbs (Fig. 3c, d). Analysis of spinal cord sections 3 days post injury revealed significantly less hemorrhaging in spinal cords from B1 B cell-depleted mice compared to controls, and this correlated with significantly less IgM and C3d deposition (Figs. 3f, g, Fig. 4). Taken together, the data demonstrate that SCI is propagated in large part by peritoneal-derived B cell natural IgM and its recognition of annexin IV and phospholipid neoepitopes in the injured spinal cord that subsequently activate complement. Further, the recognition of tissue and enhanced injury is neoepitope specific.

### Targeting complement inhibition to IgM recognition sites in the injured spinal cord

**Fig. 5:**
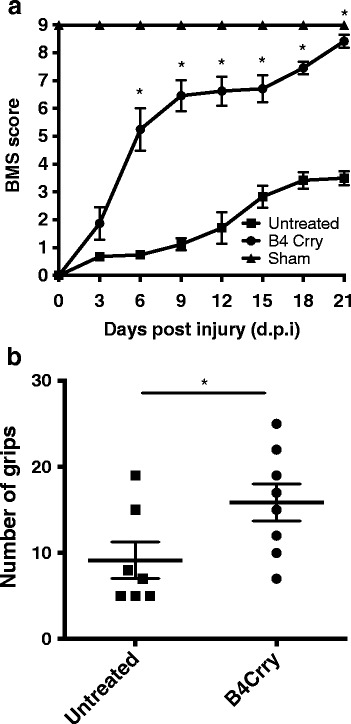
Neoepitope-targeted complement inhibition with B4-Crry improves functional outcome after SCI. **a** Assessment of locomotor activity over a 21-day period post-SCI using the 10-point BMS scale. A score of 9 represents no impairment of locomotor activity. Groups consisted of untreated injured WT C57bl/6 mice and B4-Crry-treated mice. Mean ± SE, *p < 0.001, *n* = 6–8. **b** Grip Walk test performed at 21 days post-SCI. Groups as above. Mean ± SE, *p < 0.05, *n* = 6–8

**Fig. 6:**
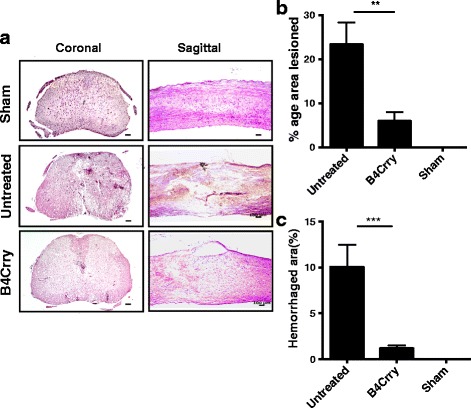
Histopathology of spinal cords from B4-Crry treated showed reduced spread of lesion 72 h after SCI. **a** H&E stained coronal and sagittal sections from epicenter of contusion injury after B4-Crry or PBS treatment. Representative images, *n* = 6. **b**, **c** Quantification of lesion and hemorrhage. Lesion size and hemorrhaged area were determined from step sections obtained across 1-mm distance from the epicenter and expressed as percentage of total area of each section analyzed. Mean ± SE, **p < 0.01, ***p < 0.001, *n* = 6.

On the basis of the above data and previous data showing both efficacy and safety benefits of site targeted complement inhibition [17, 23], we investigated whether the B4 neoepitope is a therapeutic target for complement inhibition post-SCI. We recently described the construction and functional characterization of B4-Crry, a fusion protein consisting of a scFv derived from B4 mAb linked to the mouse complement inhibitor Crry [17]. Administration of B4-Crry to WT mice 30 min after SCI significantly improved locomotor activity compared to vehicle-treated mice, and locomotor functional activity was restored by day 21 in B4-Crry-treated mice (Fig. 5a). B4-Crry treatment also significantly improved performance on a grip walk test, indicating improved restoration of sensorimotor function (Fig. 5b). The percentage lesion area per section was averaged over step sections obtained across 1 mm around the epicenter. We found significant reduction in lesion size and hemorrhaged area with B4-Crry treatment (Fig. 6).

### Effect of B4-Crry on IgM deposition and complement activation in the injured spinal cord

**Fig. 7:**
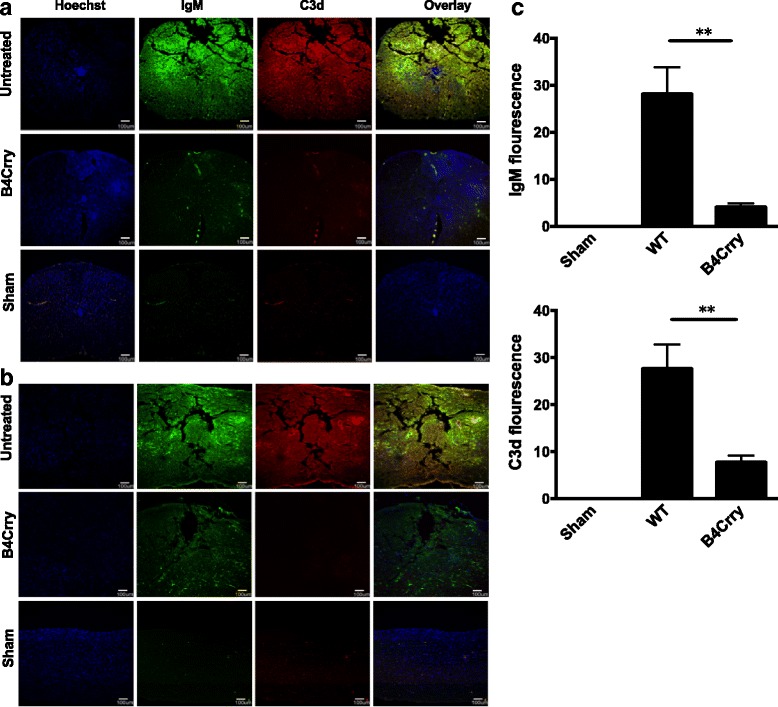
B4-Crry treatment reduces both IgM binding and C3d deposition after SCI. Analysis of of IgM and C3d deposition in vehicle treated, B4-Crry treated, and in sham operated animals at 72 h after SCI. Composite image (merged) indicates co-localization. **a** Representative coronal sections from epicenter of contusion injury. **b** Representative sagittal sections showing rostrocaudal deposition. **c** Quantification of C3d and IgM fluorescence. Quantification of IgM binding and C3d deposition was done by measuring intensity from step sections across the length of 1 mm, centered at the epicenter and starting at 100 μm from the epicenter on each side

Immunoflourescence microscopy of spinal cord sections demonstrated that B4-Crry reduced IgM binding and abolished C3d deposition at the injury epicenter (Fig. 7a), as well as rostrocaudally as indicated by analysis of longitudinal sections (Fig. 7b). Quantification of both IgM and C3d fluorescence signal suggested that the animals treated with B4Crry had significant decrease in both these signals 3 days post injury (Fig. 7c). These data indicate a dual functionality of B4-Crry in providing protection from SCI, since it not only blocks complement activation, but also inhibits the binding of endogenous pathogenic self-reactive IgM in the context of a full natural Ab repertoire.

## Discussion

Natural Abs are present from birth and are germline encoded without a requirement for exogenous antigen exposure, and it is increasingly recognized that subsets of natural Abs have varying and distinct roles. Natural IgM Abs can have homeostatic roles, for example by recognizing DAMPs expressed on stressed or dying cells and facilitating their clearance, and they can serve protective roles by a variety of cellular mechanisms [13]. However, they can also have pathogenic roles as has been shown in models of ischemia and reperfusion injury where pathogenic levels of IgM bind to ischemic tissue and activate complement [11].

It has been shown previously that following murine SCI, B cells become activated and produce pathogenic autoantibodies that propagate secondary injury in the spinal cord and impair recovery. Precisely, how traumatic injury to the spinal cord results in B cell dysfunction and autoantibody production is not clear, but pathology is caused, at least in part, via complement activation and Fc receptor ligation [6]. However, while there is accumulating evidence that B cell activation and autoantibody production play an important role in propagating SCI (reviewed in [24]), the mechanisms by which secondary spinal injury is initiated and therein the functional significance of self-reactive natural IgM Abs (as opposed to autoreactive Abs resulting from a pathogenic adaptive immune response) in this process have not been investigated. Here, we show a role for a specific subset of natural IgM Abs in activating complement and propagating secondary injury after trauma to the spinal cord and show that SCI mimics the pathological paradigm of IgM-mediated IRI in other tissues. Thus, in addition to a role for an adaptive autoantibody response in contributing to post-SCI pathology, we demonstrate a role for pre-formed self-reactive IgM Abs in an innate immune response to SCI.

Identification of the human counterpart of murine B1 B cells has been controversial, partly because CD5 is an activation marker on human B cells. However, studies have identified CD20^+^CD27^+^CD43^+^ B cells as the human B1 cell equivalent [25, 26]. Although B1 cells had been thought to be located predominantly in the peritoneal and pleural cavities of mice, recent evidence indicates that the bone marrow and spleen may be an important source [27]. Nevertheless, the current study indicates that peritoneal B1 cell-derived natural IgM plays an important role in propagating SCI, since peritoneal depletion of B1 B cells both significantly reduced levels of circulating pathogenic IgM of B4 and C2 specificity and significantly improved recovery. Both our data and previous studies have shown that these B1 depletion procedures do not alter total IgM levels [28]. Together, these data suggest a role for distinct subsets of naturally occurring IgM Abs and raise the possibility of different Ab repertoires originating from different B1 cell locations.

The B4 and C2 IgM mAbs that we utilize in this study have been previously described: B4 recognizes a post-translational modification of annexin IV [9] and C2 recognizes a subset of phospholipids [10].The natural Ab status of these mAbs is indicated by the fact that they were derived from unmanipulated mice, they are universally present in C57BL/6 mice, and the circulatory levels of IgM Abs of B4 and C2 specificity are reduced following peritoneal B1 cell depletion. Also, natural IgM Ab recognition of neoepitopes is the accepted paradigm for complement activation following ischemia and reperfusion of other tissues. We show here that the B4 and C2 antigens represent DAMPs expressed in the injured spinal cord. Injury was contingent upon specific recognition of the injured spinal cord, since unlike B4 and C2 mAbs, control IgM mAbs, including one that was isolated from the same panel containing the B4 and C2 mAbs, neither bound within the injured spinal cord nor reconstituted injury in otherwise protected Ab-deficient Rag1−/− mice. In an earlier report, Rag2−/− mice were shown to have improved recovery following spinal cord contusion compared to wt mice, with improved locomotor function and increased axonal regrowth [29]. Improved recovery in Rag2−/− mice was associated with decreased microglia/macrophage infiltration, which the authors attributed to the absence of T cell-derived cytokines. Certainly, this could be a contributing mechanism of injury, besides the critical role for natural Abs in activating complement and initiating injury.

It is interesting that specific natural IgM Abs are also implicated in CNS repair mechanisms, beyond their role in identifying injured and dying cells for removal, and some IgM mAbs have shown potential for therapeutic application following CNS injury (reviewed in [16]). These protective Abs appear to function by providing intracellular signals that promote neuron or glial cell survival. The mechanistic relationship between pathogenic and protective natural Abs is not clear. There may be quantitative and/or qualitative, as well as temporally acting or context-dependent differences between Abs that are involved in injury vs. repair. It is also noteworthy that complement activation products can have both neurodegenerative and neuroregenerative effects following CNS injury [30, 31]. While complement-mediated protective mechanisms likely become more important in the subacute phase after CNS injury [32], this balance between complement-mediated injury and repair is an area in need of further investigation.

Based on the identification of neoepitopes exposed in the injured spinal cord and the pathogenic and complement-activating properties of mAbs recognizing these neoepitopes, we investigated a site-targeted therapeutic strategy for the local delivery of a complement inhibitor. Site-targeted complement inhibition obviates the need for systemic inhibition, increases bioavailability, and markedly increases efficacy (over 10-fold) [23]. We have also shown previously that unlike systemic inhibitors, therapeutic doses of site-targeted complement inhibitors do not adversely affect important physiological functions such as host defense [17, 23]. This latter point is an important consideration for the treatment of SCI patients, since CNS injury is immunosupressive and there is a high incidence of morbidity and mortality caused by infection in neurotrauma and SCI patients.

In our therapeutic paradigm, we used our recently described fusion protein consisting of a single chain (scFv) derived from the B4 hybridoma linked to Crry, a murine orthologue of human sCR1 [17]. In our previous report, we also demonstrated that B4-Crry does not increase susceptibility to infection [23, 33]. Here, the protective effect of B4-Crry correlated not only with significantly reduced complement activation, but also reduced IgM deposition at the injury site. This indicates that in addition to complement inhibition, B4-Crry is inhibiting the binding of IgM Abs to the injured spinal cord in the context of a full natural Ab repertoire. This could be due to B4 scFv competing for IgM binding sites, but also due to decreased complement-mediated injury with a reduction in the associated exposure of neoepitopes. While it can be expected that there is much redundancy in IgM and neoepitope specificities, we have nonetheless shown that B4 scFv alone can significantly reduce IgM binding in the post-ischemic heart [17]. A possible explanation for this effect of a single scFv specificity on overall IgM binding is that there is a requirement for recognition of exposed neoepitopes in a chronological sequence, such that disrupting any one of these interactions ameliorates complement activation. Another possibility is that recognition of these neoepitopes on multiple cell types is critical for complement dependent injury, and blocking even one of these specificities is consequential for the overall complement mediated injury response.

## Conclusions

This study identifies important neoepitopes expressed within the spinal cord after injury, that are recognized by clonally specific natural IgM Abs that activate complement and drive secondary injury. Peritoneal B1 cells are a source of a subset of pathogenic self-reactive Abs that contribute to pathology by activating complement. Post-SCI expressed neoepitopes represent novel targets for the targeted delivery of therapeutics to the injured spinal cord, and we show that targeted delivery of a complement inhibitor significantly improves recovery following trauma to the spinal cord. With regard to the potential translation of this therapeutic strategy to the clinic, it has been shown that the B4 annexin IV neoepitope is also expressed on stressed and injured human cells [10]. Therefore, this antibody based targeting strategy presents a new therapeutic payload delivery option in an injury site specific manner.

## Additional file

Additional file 1: Figure S1. (A) Spinal cord tissue analyses were done across the length of 1 mm centered at the epicenter (B). Examples of hemorrhaged (red) and lesioned (gray) area are outlined. The outlined area for each section measured using a Nikon eclipse E600 with axiovision outline tool and was expressed as a percentage of total area of each section measured. **Figure S2.** (A) After B1 cell depletion procedure, the peritoneal fluid was analyzed by flow cytometry to confirm a decrease in CD19^+^IgM^hi^B220^lo^ populations. Initial gate was placed on live cells and viability determined by 7-AAD (7-Aminoactinomycin D) staining followed by gating on CD19+ and IgMhi cells. The B220^lo^ subpopulation from the gated population was used for comparison among different groups (B). The mice that received PBS injections as control were used for these analyses. *n* = 6. (PDF 843 kb)

## Abbreviations

Ab: Antibody
IRI: Ischemia reperfusion injury
mAb: Monoclonal antibody
scFv: Single-chain antibody
SCI: Spinal cord injury
