# Supplementary material for: Natural IgM antibodies that bind neoepitopes exposed as a result of spinal cord injury , drive secondary injury by activating complement
Source: J Neuroinflammation. 2017 Jun 19;14:120. doi: 10.1186/s12974-017-0894-6 (PMC5477255; doi:10.1186/s12974-017-0894-6)
Supplement: Additional file 1: Figure S1. — (A) Spinal cord tissue analyses were done across the length of 1 mm centered at the epicenter (B). Examples of hemorrhaged (red) and lesioned (gray) area are outlined. The outlined area for each section measured using a Nikon eclipse E600 with axiovision outline tool and was expressed as a percentage of total area of each section measured. Figure S2. (A) After B1 cell depletion procedure, the peritoneal fluid was analyzed by flow cytometry to confirm a decrease in CD19+IgMhiB220lo populations. Initial gate was placed on live cells and viability determined by 7-AAD (7-Aminoactinomycin D) staining followed by gating on CD19+ and IgMhi cells. The B220lo subpopulation from the gated population was used for comparison among different groups (B). The mice that received PBS injections as control were used for these analyses. n = 6. (PDF 843 kb) [file 12974_2017_894_MOESM1_ESM.pdf]

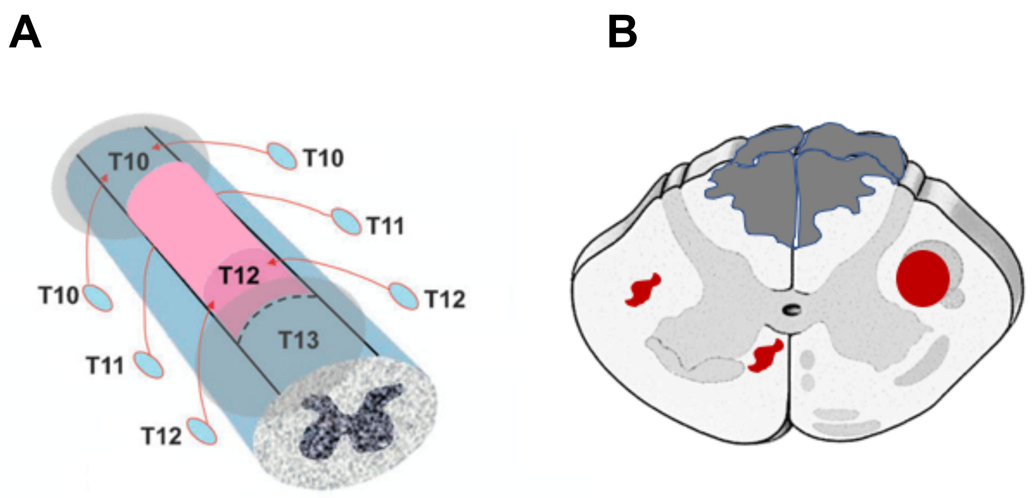

Figure S1: (A) Spinal cord tissue analyses were done across the length of 1mm centered at the epicenter (B). Examples of hemorrhaged (red) and lesioned (gray) area are outlined. The outlined area for each section measured using a Nikon eclipse E600 with axiovision outline tool, and was expressed as a percentage of total area of each section measured.

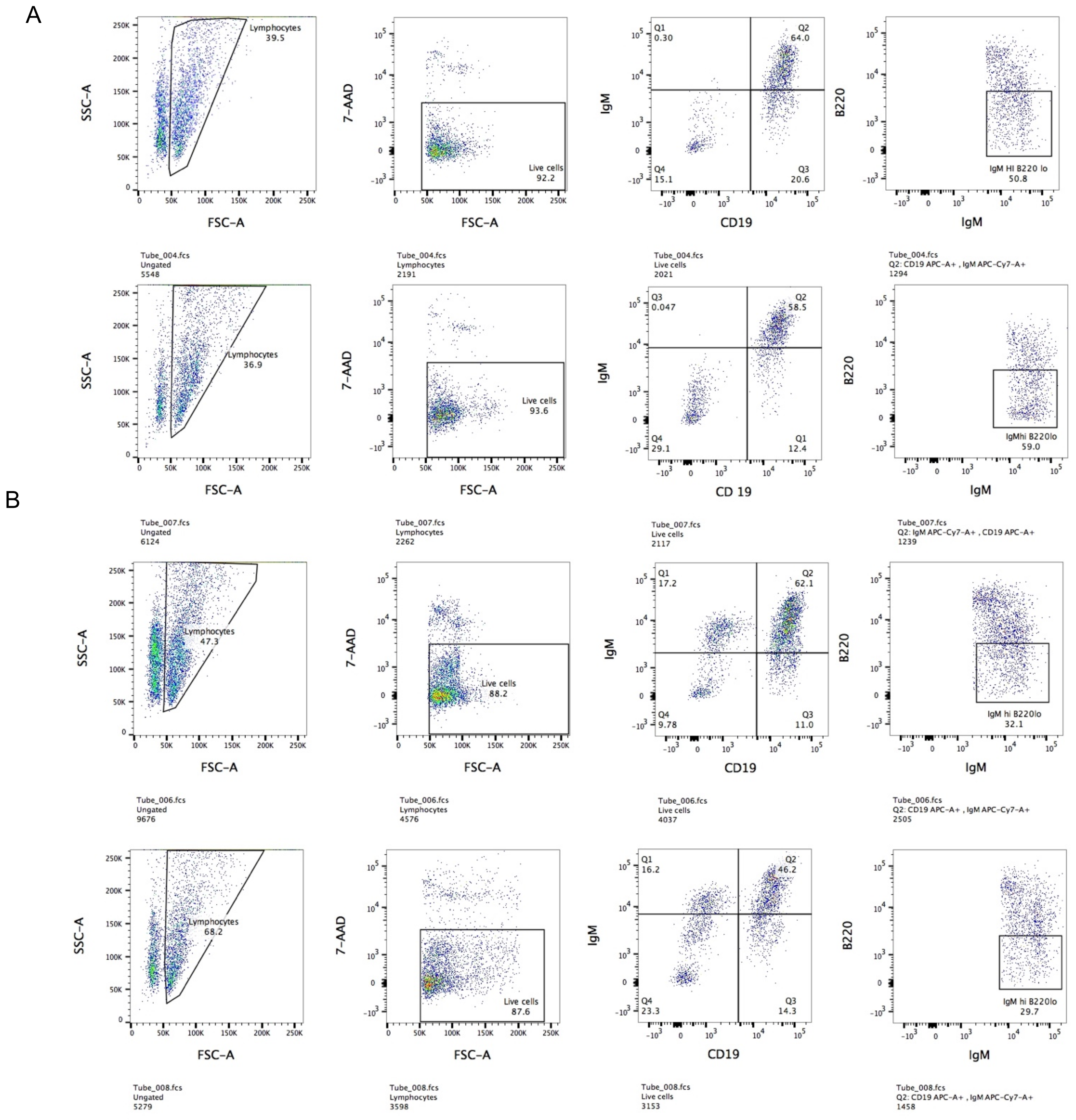

Figure S2: (A) After B1 cell depletion procedure, the peritoneal fluid was analyzed by flow cytometry to confirm a decrease in $CD19^{+}IgM^{hi}B220^{lo}$ populations. Initial gate was placed on live cells and viability determined by 7-AAD (7-Aminoactinomycin D) staining followed by gating on CD19+ and IgM^{hi} cells. The $B220^{lo}$ subpopulation from the gated population was used for comparison among different groups (B) The mice that received PBS injections as control were used for these analyses. n=6
